# Atomic-scale diffusion rates during growth of thin metal films on weakly-interacting substrates

**DOI:** 10.1038/s41598-019-43107-8

**Published:** 2019-04-29

**Authors:** A. Jamnig, D. G. Sangiovanni, G. Abadias, K. Sarakinos

**Affiliations:** 1 Institut Pprime, Département Physique et Mécanique des Matériaux, UPR 3346 CNRS, Université de Poitiers, SP2MI, 11 Bvd M. et P. Curie, F 86073 Poitiers Cedex 9, France; 2 Nanoscale Engineering Division, Department of Physics, Chemistry, and Biology, Linköping University, SE 581 83 Linköping, Sweden; 3 Atomistic Modelling and Simulation, ICAMS, Ruhr-Universität Bochum, D-44801 Bochum, Germany; 4 Theoretical Physics Division, Department of Physics, Chemistry, and Biology, Linköping University, SE 581 83 Linköping, Sweden

**Keywords:** Surfaces, interfaces and thin films, Design, synthesis and processing

## Abstract

We use a combined experimental and theoretical approach to study the rates of surface diffusion processes that govern early stages of thin Ag and Cu film morphological evolution on weakly-interacting amorphous carbon substrates. Films are deposited by magnetron sputtering, at temperatures *T*_*S*_ between 298 and 413 *K*, and vapor arrival rates *F* in the range 0.08 to 5.38 *monolayers*/*s*. By employing *in situ* and real-time sheet-resistance and wafer-curvature measurements, we determine the nominal film thickness Θ at percolation (Θ_*perc*_) and continuous film formation (Θ_*cont*_) transition. Subsequently, we use the scaling behavior of Θ_*perc*_ and Θ_*cont*_ as a function of *F* and *T*_*s*_, to estimate, experimentally, the temperature-dependent diffusivity on the substrate surface, from which we calculate Ag and Cu surface migration energy barriers ${{\boldsymbol{E}}}_{{\boldsymbol{D}}}^{{\bf{\exp }}}$ and attempt frequencies ${{\boldsymbol{\nu }}}_{{\bf{0}}}^{{\bf{\exp }}}$. By critically comparing ${{\boldsymbol{E}}}_{{\boldsymbol{D}}}^{{\bf{\exp }}}$ and ${{\boldsymbol{\nu }}}_{{\bf{0}}}^{{\bf{\exp }}}$ with literature data, as well as with results from our *ab initio* molecular dynamics simulations for single Ag and Cu adatom diffusion on graphite surfaces, we suggest that: (i) ${{\boldsymbol{E}}}_{{\boldsymbol{D}}}^{{\bf{\exp }}}$ and ${{\boldsymbol{\nu }}}_{{\bf{0}}}^{{\bf{\exp }}}$ correspond to diffusion of multiatomic clusters, rather than to diffusion of monomers; and (ii) the mean size of mobile clusters during Ag growth is larger compared to that of Cu. The overall results of this work pave the way for studying growth dynamics in a wide range of technologically-relevant weakly-interacting film/substrate systems—including metals on 2D materials and oxides—which are building blocks in next-generation nanoelectronic, optoelectronic, and catalytic devices.

## Introduction

The fabrication of heterostructure devices founded upon weakly-interacting 2D-material (e.g., graphene and MoS_2_) and oxide (e.g., TiO_2_ and ZnO) substrates largely relies on the essential step of growing thin metal films with controlled morphology, to serve as electrical contacts, or active optical and catalytic layers^1–4^. Such films are typically synthesized via condensation from the vapor phase, and their morphology is, predominantly, governed by the occurrence rates of atomic-scale surface diffusion processes during early growth stages^5^. These rates are not well-established in the literature—as opposed to the case of metal-on-metal homoepitaxial growth^6–9^—and they are estimated indirectly, either by post-deposition *ex situ* analyses of island densities and sizes using electron and atomic force microscopies^10–12^, or by studying de-wetting of continuous metal layers upon annealing^12,13^.

Lü *et al*.^14^ have recently suggested a method for determining, *in situ* and in real time, the effective atomic-scale surface diffusivity during metal film growth on weakly-interacting substrates, by using scaling relations of the nominal film thickness Θ at characteristic morphological transitions (elongation, percolation and continuous film formation) with respect to the deposition rate *F*. They also argued that the accuracy of this method can be further improved by studying the way by which the deposition temperature *T*_*S*_ affects film morphological evolution, as this would allow to gauge the effect of surface vibrations on diffusion dynamics.

In the present work, we use the method originally presented in ref.^14^ to study growth of sputter-deposited Ag and Cu films on weakly-interacting amorphous carbon (a-C) substrates, for *T*_*S*_ between 298 and 413 *K*, and *F* in the range 0.08 to 5.38 *monolayers*/*s*(*ML*/*s*). We determine Θ at percolation (Θ_*perc*_) and continuous film formation (Θ_*cont*_) transitions by means of *in situ* and real-time sheet-resistance and wafer-curvature measurements, respectively. Using the scaling behavior of Θ_*perc*_ and Θ_*cont*_ as function of *F* and *T*_*S*_, we estimate a temperature-dependent Ag diffusivity on the substrate surface *D*_*Ag*_(*T*_*S*_), which is up to three orders of magnitude larger than the diffusivity of Cu *D*_*Cu*_(*T*_*S*_). Linear regression analysis of ln(*D*_*Ag*_(*T*_*S*_)) and ln(*D*_*Cu*_(*T*_*S*_)) vs. 1/*T*_*S*_ data enables us to extract Ag and Cu surface diffusion energy barriers ${E}_{D}^{exp}$ and attempt frequencies ${\nu }_{0}^{exp}$. We also perform *ab initio* molecular dynamics (AIMD) simulations—within the framework of density functional theory (DFT)—of single Ag and Cu adatom diffusion on graphite surface for *T*_*S*_ = 300–1000 *K*, from which we compute adatom surface migration energy barriers and attempt frequencies. By critically comparing ${E}_{D}^{AIMD}$ and ${\nu }_{0}^{AIMD}$ with their respective experimental values, as well as with literature data, we suggest that: (i) $\,{E}_{D}^{exp}$ and ${\nu }_{0}^{exp}$ correspond to metal cluster diffusion on a-C substrates, which is the rate limiting process for determining the early-stage Ag and Cu film morphology, and (ii) the mean size of mobile Ag clusters is larger than that of Cu clusters. The overall results of this work are the first step toward determining diffusion rates in film/substrate systems which are relevant for device fabrication in the areas of nanoelectronics, catalysis, and architectural glazing^3,15^.

The paper is organized as follows: First, we present a brief theoretical background concerning the scaling laws of Θ at characteristic morphological transitions during growth of metals on weakly-interacting substrates and we discuss the way by which these laws are used to calculate atomic surface diffusion rates. Then, experimental and simulation results are presented and discussed. Finally, the overall study is summarized. The section “Methods” outlines the experimental procedures employed in this study and provides a description of the methodology used for DFT-based calculations and simulations.

## Morphological Transitions and their Scaling Behavior

Thin film growth starts with nucleation of isolated islands, which grow in size, until they impinge on each other and start to coalesce. As island size increases further, with continued deposition and coalescence, material redistribution among islands becomes progressively slow, which eventually stops the process of coalescence and leads to the formation of a percolated network of interconnected islands separated by voids. Further deposition fills the inter-island space until a continuous film is formed.

Metal islands deposited on weakly-interacting substrates typically exhibit a pronounced 3D shape^16–18^. The time *τ*_*coal*_ required for completion of coalescence of a pair of such islands—i.e., the time from island impingement until the equilibrium shape of the island pair is established—can be approximated by the classical expression developed by Nichols^19^ for sintering as ${\tau }_{coal}=\frac{{R}^{4}}{B}$; where *R* is the radius of the smaller island in the coalescing pair, and *B* is the coalescence-rate parameter that scales with the adatom self-diffusivity *D*_*s*_. According to the expression ${\tau }_{coal}=\frac{{R}^{4}}{B}$, as *R* increases, *τ*_*coal*_ increases as well, and there is a critical *R* value at which *τ*_*coal*_ becomes longer than the time required for a third island to impinge on a coalescing island pair. This point during growth corresponds to the so-called *elongation transition*, beyond which the film surface consists predominantly of elongated non-coalesced groups of islands.

Analytical modelling, based on the droplet growth theory^20,21^, and kinetic Monte Carlo simulations^22–26^ suggest that, for film materials and deposition parameters for which coalescence is the dominant process during early stages of film growth (*coalescence-controlled regime*), the nominal film thickness at the elongation transition Θ_*elong*_ scales with *F* as

1 $${{\rm{\Theta }}}_{elong} \sim {(\frac{B}{F})}^{\frac{1}{3}}.$$

This expression encodes the effect of dynamic competition among island growth and coalescence on film morphological evolution. For a constant coalescence-rate parameter *B*, increase of *F* yields a larger island growth rate, so that an elongated surface morphology is attained at smaller nominal thicknesses. Conversely, increase of *B*, at a constant *F*, promotes coalescence completion relative to island growth, thereby delaying the occurrence of elongation transition.

For a given film/substrate system, one can determine deposition conditions in terms of *F* and *T*_*S*_, for which coalescence is not completed throughout all stages of growth (*coalescence-free regime*)^23,27^. In this case, Θ_*elong*_ becomes proportional to the island-island separation distance when island density reaches saturation N_*sat*_^22,28^, i.e., ${{\rm{\Theta }}}_{elong} \sim {{{\rm{N}}}_{sat}}^{-\frac{1}{2}}$. From the atomistic nucleation theory^29^, N_*sat*_ for 3D growth is calculated by the expression ${{\rm{N}}}_{sat} \sim {(\frac{F}{D})}^{\frac{2}{7}}$, which yields

2 $${{\rm{\Theta }}}_{elong} \sim {(\frac{D}{F})}^{\frac{1}{7}}.$$

We note here that the expression ${{\rm{N}}}_{sat} \sim {(\frac{F}{D})}^{\frac{2}{7}}$ holds for a critical cluster size i* = 1 (i* is expressed in number of atoms)—i.e., for the case of a dimer being the smallest stable island on the substrate surface—and for immobile clusters^30^. The validity of the condition i* = 1 and the implications of cluster mobility for the conclusions drawn in the present study are elaborated upon in the “Discussion” section.

The adatom surface diffusivity *D* in Eq. (2) is equal to

3 $$D={D}_{0}\,\exp \,(-\frac{{E}_{D}}{{k}_{B}{T}_{S}}).$$

The prefactor *D*_0_ can be approximated as ${D}_{0}=\frac{1}{4}{\nu }_{0}{a}^{2}$, where *ν*_0_ is the attempt frequency for adatom migration and *a* is the minimum adatom translational hopping distance on the substrate surface. *E*_*D*_ is the surface diffusion activation barrier. It should be noted that *D*_*S*_, which largely determines *B*, is also calculated from Eq. (3), by using the *E*_*D*_, *ν*_0_, and *a* values for the surface of the deposited film material. In analogy to coalescence-controlled growth (Eq. (1)), Eq. (2) reflects the way by which the interplay among island nucleation and growth affects the early-stage film morphology. Increase of *D*, for a given *F*, favors growth of existing islands, at the expense of nucleation of new ones, resulting in an increase of the nominal thickness required for the onset of island-island impingement. In the opposite case, larger *F*, for a constant *D*, promotes nucleation, and pushes elongation to occur at smaller nominal thicknesses.

The growth regimes with respect to coalescence can be identified experimentally using the following procedure: (i) establish the existence of a linear relationship between Θ_*elong*_ and 1/*F* in logarithmic scale; (ii) determine the slope of the linear function that describes Θ_*elong*_ vs. 1/*F* data; and (iii) compare this slope with the scaling exponents in Eqs (1) and (2). By repeating steps (i) through (iii) for various *T*_*S*_ values in the *coalescence-free* (*coalescence-controlled*) growth regime, temperature-dependent *D*(*T*_*S*_) (*B*(*T*_*S*_)) values can be calculated. From this, surface diffusion (self-diffusion) energy barriers and attempt frequencies can be extracted via linear regression on ln(*D*(*T*_*S*_)) (ln(*B*(*T*_*S*_))) vs. 1/*T*_*S*_ data. It should be noted that the elongation transition is an intrinsically abstract concept, i.e., Θ_*elong*_ is difficult to determine experimentally^14^. Hence, the scaling behavior in view of Eqs (1) and (2) is, typically, studied using later morphological transition thicknesses, i.e., Θ_*perc*_ and Θ_*cont*_^23,24^, which have been shown to scale linearly with Θ_*elong*_^14,23,31^. In the present study, we implement the above-explained procedure for determining surface migration energy barriers and attempt frequencies during film growth of Ag and Cu on a-C. We employ sputter deposition to access multiple growth regimes and establish the Θ_*perc*_ and Θ_*cont*_ vs. 1/*F* relationships at multiple *T*_*S*_ values.

## Results

### Film morphological transitions and evolution

**Figure 1:**
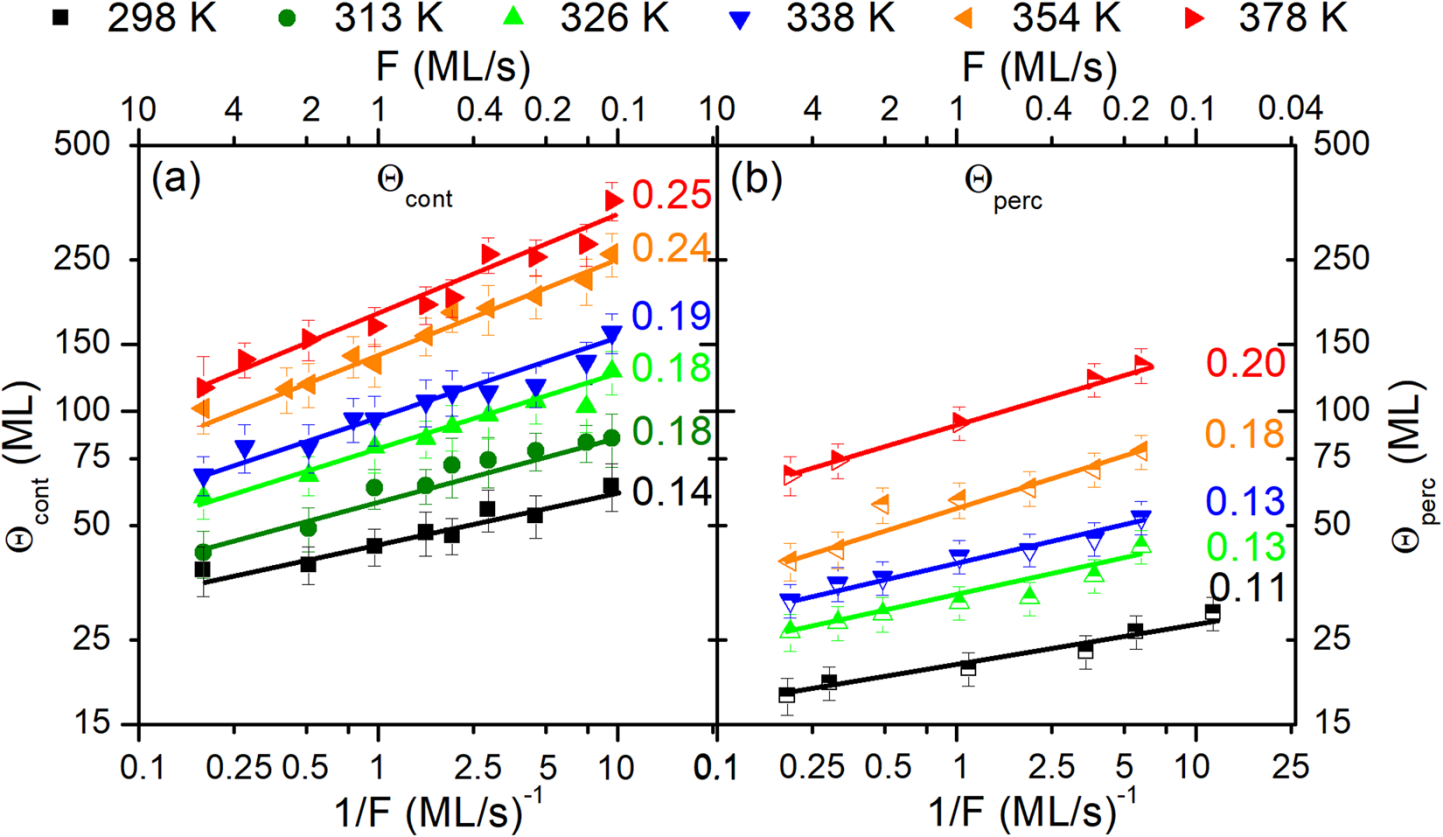
Evolution of (**a**) continuous film formation thickness *Θ*_*cont*_ and (**b**) percolation thickness *Θ*_*perc*_ during growth of Ag on amorphous carbon as a function of inverse deposition rate 1/*F* at various growth temperatures *T*_*S*_. *F* values are presented in the top axis for clarity. Error bars correspond to the standard error during acquisition of the (**a**) *σ* × Θ, (**b**) *R*_*S*_ vs. Θ data and the graphical determination of *Θ*_*cont*_ and *Θ*_*perc*_ values, respectively. The numbers next to each *T*_*S*_ set are the slopes of linear fits to the data points in log-log scale.

The evolution of Θ_*cont*_ vs. 1/*F* (in logarithmic scale), for the Ag/a-C film/substrate system, at *T*_*S*_ = 298–378 *K*, is plotted in Fig. 1(a). Representative curves of the film stress-thickness product (*σ* × Θ) vs. Θ, from which Θ_*cont*_ is determined, are shown in Fig. S1 in the Supplemental Material^32^. Details on the stress measurement methodology can be found in the “Methods” section. We see in Fig. 1(a) that Θ_*cont*_ varies linearly with 1/*F*, for all *T*_*S*_ values, with the slope *χ* (i.e., the exponent of the Θ_*cont*_ ~ (1/*F*)^*χ*^ function in linear scale) indicated next to each set of data. For *T*_*S*_ = 298 *K*, an exponent *χ* of 0.14 is obtained, which matches the theoretical value for *coalescence-free* growth (1/7, see Eq. (2)). Furthermore, this result is in excellent agreement with the scaling exponent found by Lü *et al*.^25^—using *in situ* spectroscopic ellipsometry—for room-temperature sputter-deposition of Ag films on SiO_2_.

Increase of the growth temperature to the two largest values of 354 and 378 *K*, yields *χ* = 0.24–0.25, which is smaller than the theoretical value of 1/3 for *coalescence-controlled* growth (see Eq. (1)). Lü *et al*.^25^ have suggested that Θ_*cont*_ values which scale as a function of 1/*F* with an exponent *χ* in the range 0.25 to 0.28 are associated with a broad transition region (spanning up to two orders of magnitude in *F*) between *coalescence-controlled* and *coalescence-free* growth regimes. In addition, Fig. 1(a) shows that *χ* = 0.18–0.19 for 313 ≤ *T*_*S*_ ≤ 338 *K*, which is also an indication that the transition between the two growth regimes occurs gradually. Finally, we note that it was not possible to determine Θ_*cont*_ for *T*_*S*_ > 378 *K*, as the intrinsic stress in the film becomes too small and the *in situ* curvature measurements unreliable.

The dependence of Θ_*perc*_ on 1/*F* is presented in Fig. 1(b) in logarithmic scale, while typical film resistivity (*R*_*S*_) vs. Θ curves, which are used for determining Θ_*perc*_, are provided in Fig. S2 in the Supplemental Material^32^. Details on the film resistivity measurement methodology can be found in the “Methods” section. For each set of *F* and *T*_*S*_ values in Fig. 1(b), Θ_*perc*_ is smaller than the corresponding Θ_*cont*_, and the two thicknesses exhibit a nearly constant $\frac{{{\rm{\Theta }}}_{perc}}{{{\rm{\Theta }}}_{cont}}$ ratio *κ* ~ 0.5 at all deposition conditions. The relationship Θ_*perc*_ < Θ_*cont*_ is consistent with the growth evolution stages explained in the “Morphological transitions and their scaling” section; a continuous layer is formed when deposited material fills the inter-island space of a percolated film.

Analysis of the scaling behavior Θ_*perc*_ ~ (1/*F*)^*χ*^ shows that, at *T*_*S*_ = 298 *K*, a *χ* value of 0.11 is obtained, which gradually increases to *χ* = 0.2 upon increasing temperature to *T*_*S*_ = 378 *K*. Moreover, we see that the *χ* values for Θ_*perc*_ are slightly smaller than the corresponding exponents for Θ_*cont*_. Despite the small quantitative differences in the scaling exponents of Θ_*perc*_ and Θ_*cont*_ vs. 1/*F*, the overall asymptotic qualitative trends in Fig. 1 indicate a transition from *coalescence-free* toward *coalescence-controlled* growth with increasing *T*_*S*_.

**Figure 2:**
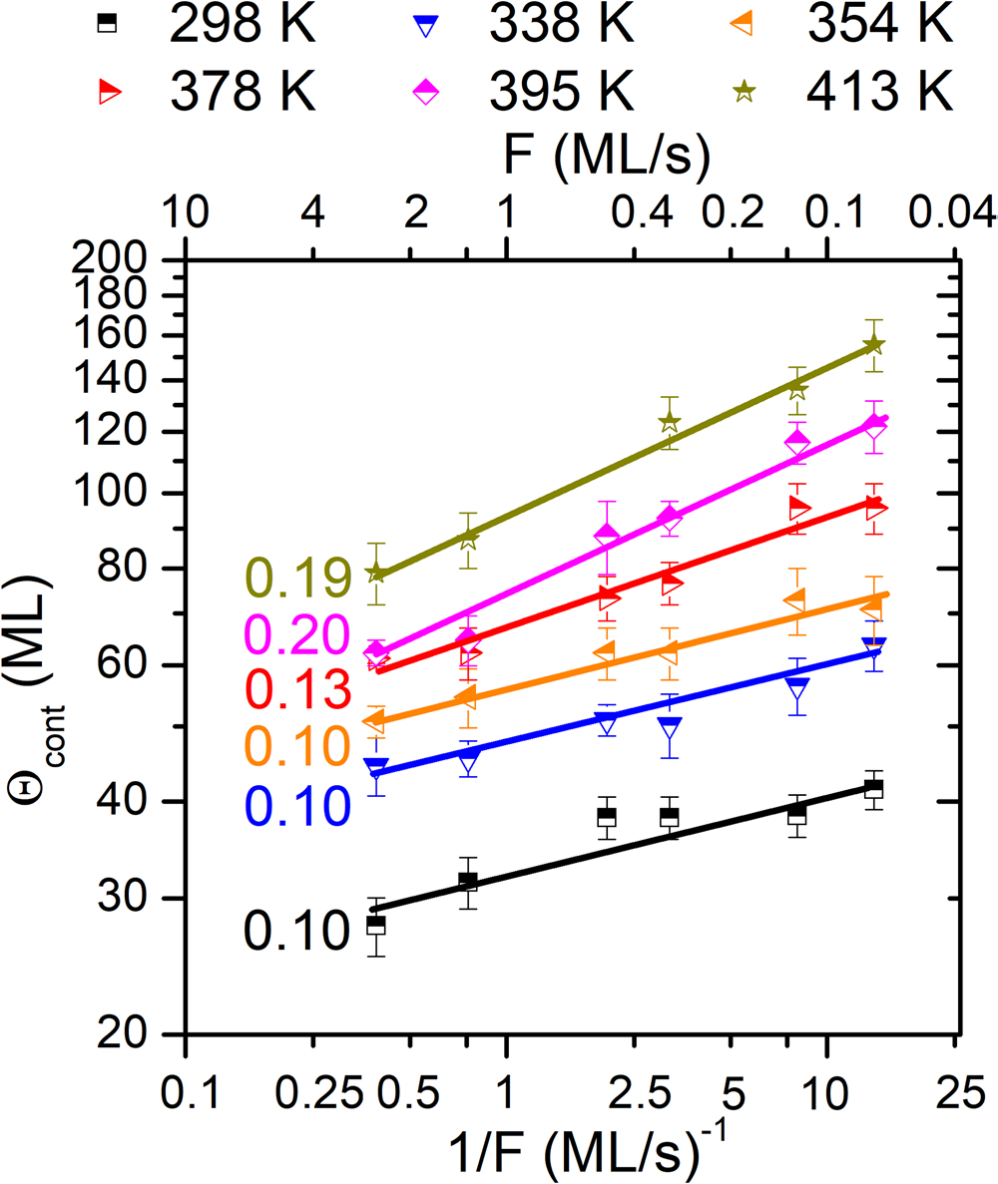
Evolution of continuous film formation thickness *Θ*_*cont*_ during growth of Cu on amorphous carbon as a function of inverse deposition rate 1/*F* at various growth temperatures *T*_*S*_. *F* values are presented in the top axis for clarity. Error bars correspond to the standard error during acquisition of the *σ* × Θ vs. Θ data and the graphical determination of the *Θ*_*cont*_ values. The numbers next to each *T*_*S*_ set are the slopes of linear fits to the data points in log-log scale.

*In situ* and real-time wafer-curvature measurements were also employed during Cu growth on a-C (see Fig. S3 in Supplemental Material^32^ for representative *σ* × Θ vs. Θ curves), from which Θ_*cont*_ was determined. Θ_*cont*_ vs. 1/*F* data are plotted in Fig. 2 for *T*_*S*_ in the range 298 to 413 *K*. As in the Ag/a-C system, a linear relationship between Θ_*cont*_ and 1/*F* is observed, and the corresponding slopes *χ* are given next to each dataset. The scaling exponent *χ* equals 0.10 for *T*_*S*_ = 298–354 *K*, and increases slightly to 0.13 for *T*_*S*_ = 378 *K*. For temperatures above 378 *K*, *χ* takes a value of 0.19–0.20. The evolution of *χ* vs. *T*_*S*_ described above is qualitatively similar to that of Ag, and supports the fact that an increase in *T*_*S*_ favors *coalescence-controlled* growth.

Comparison between Figs 1(a) and 2 shows that Θ_*cont*_ for Cu lies in the range 25 to 150 *ML*, which is smaller than the corresponding values determined for Ag (40 *ML* < Θ_*cont*_ < 360 *ML*). These differences imply that, at the deposition conditions used in the present work, nucleation (coalescence) rates are smaller (larger) for Ag compared to Cu, leading to a more pronounced 3D morphology in the Ag/a-C system.

We also determined Θ_*perc*_ from *in situ* and real-time sheet-resistance measurements for Cu, for selected *F* values and *T*_*S*_ ≤ 354 *K*. The results (not presented here) reveal that Θ_*perc*_ < Θ_*cont*_ with $\kappa \,=\frac{{{\rm{\Theta }}}_{perc}}{{{\rm{\Theta }}}_{cont}}\sim 0.3$, while the Θ_*perc*_ ~ (1/*F*)^*χ*^ scaling exponent *χ* is equal to 0.07–0.08. These values are qualitatively consistent with the results for the Ag/a-C system, where *χ* for Θ_*perc*_ was found to be lower than the corresponding value for Θ_*cont*_. The overall results for Θ_*perc*_ and Θ_*cont*_ indicate that the morphological evolution of Cu films on a-C proceeds in the *coalescence-free* growth regime for *T*_*S*_ ≤ 378 *K*.

**Figure 3:**
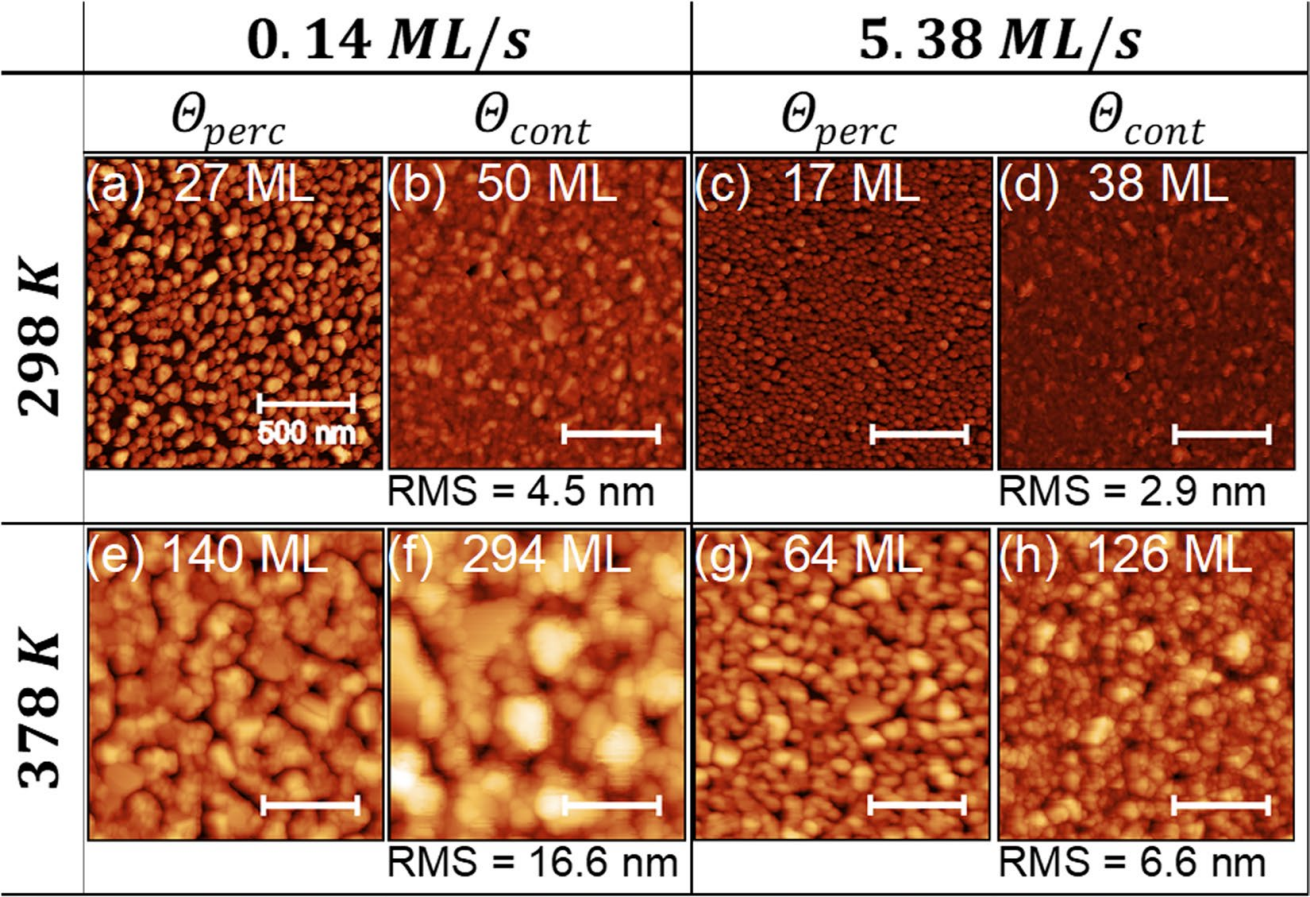
Atomic force microscopy images of the surface morphology of Ag grown on amorphous carbon at deposition temperatures *T*_*S*_ of 298 *K* ((**a**–**d**)) and 378 *K* ((**e**–**h**)) and deposition rates *F* of 0.14* ML*/*s* ((**a**,**b**,**e**,**f**)) and 5.38 *ML*/*s* ((**c**,**d,g,h**)). For each pair of deposition parameters *T*_*S*_ and *F*, images of films at percolation *Θ*_*perc*_ ((**a**,**c**,**e**,**g**)) and continuous film formation *Θ*_*cont*_ thicknesses ((**b**,**d**,**f**,**h**)) are presented. The nominal thickness is indicated in the respective figures, and root-mean-square roughness (RMS) values are given for the continuous films. The height scale is 60 *nm* for images (**a**–**d**), (**g**,**h**), and 120 *nm* for images (**e**,**f**).

In order to confirm the film morphological transitions and evolution inferred by the *in situ* data in Figs 1 and 2, we studied *ex situ* the film surface topography by atomic force microscopy (AFM). Figure 3 presents AFM images recorded for Ag films grown on a-C at Θ_*perc*_ and Θ_*cont*_, and at the following sets of conditions: (i) *T*_*S*_ = 298 *K* and *F* = 0.14 *ML*/*s* (Fig. 3(a,b)), (ii) *T*_*S*_ = 298 *K* and *F* = 5.38 *ML*/*s* (Fig. 3(c,d)), (iii) *T*_*S*_ = 378 *K* and *F* = 0.14 *ML*/*s* (Fig. 3(e,f)), and (iv) *T*_*S*_ = 378 *K* and *F* = 5.38 *ML*/*s* (Fig. 3(g,h)). At all conditions imaged in Fig. 3, the fraction of the substrate area covered by the Ag films is larger at Θ_*cont*_, as compared to Θ_*perc*_, which provides further support to the fact that the experimentally determined values of Θ_*perc*_ and Θ_*cont*_ in Figs 1 and 2 are consistent with the different stages of film morphological evolution.

Moreover, Fig. 3 shows that, for a given deposition rate, increase of *T*_*S*_ from 298 *K* to 378 *K* leads to an increase of the size of the features (i.e., islands) on the film surface, compare, e.g., Fig. 3(b) vs. (f) and Fig. 3(d) vs. (h). These differences in morphology translate into changes of the surface roughness, e.g., the root-mean-square roughness (RMS) value of a Ag film at Θ_*cont*_, grown with *F* = 0.14 *ML*/*s*, increases from 4.5 to 16.6 *nm* when *T*_*S*_ is increased from 298 to 378 *K* (Fig. 3(b) vs. (f)). Conversely, increase of *F* for a given growth temperature leads to smoother films. As an example, the RMS roughness value of 16.6 *nm* of a Ag film at Θ_*cont*_, grown with *F* = 0.14 *ML*/*s* and at *T*_*S*_ = 378 *K* (Fig. 3(f)) decreases to 6.6 *nm*, when increasing *F* to 5.38 *ML*/*s* for the same growth temperature (Fig. 3(h)).

AFM measurements were also performed for Cu films grown on a-C, and the results were again found consistent with the *in situ* analysis data. Cu shows a smoother surface morphology, as compared to Ag, for similar deposition conditions, (see Fig. S4 in the Supplemental Material^32^). At Θ_*cont*_, the RMS roughness of Ag (6.6 *nm*) is higher than that of Cu (1.7 *nm*), and this trend persists for nominal thicknesses of *Θ* ≅ 450 *ML*, where Ag films (10.9 *nm*) remain rougher than Cu films (6.2 *nm*).

### Atomic-scale diffusion rates

As discussed in the previous section, the results in Figs 1 and 2 indicate that increase of *T*_*S*_ causes a transition toward *coalescence-controlled* growth, which is in agreement with previously reported data by Warrender and Aziz^24^ and Lü *et al*.^25^. However, there is no set of Θ_*perc*_ and Θ_*cont*_ vs. 1/*F* data that can be clearly associated with this growth regime. Concurrently, the scaling exponent *χ* for Ag (*T*_*S*_ ≤ 338 *K*) and Cu (*T*_*S*_ ≤ 378 *K*) matches, or is in close agreement with the theoretically-predicted value of 1/7 for *coalescence-free* growth for i* = 1. Based on the arguments presented above, in the remainder of the manuscript we use data in the *coalescence-free* growth regime to determine the surface diffusivity *D*.

Previous studies in homoepitaxial Ag/Ag(100)^33^ and Cu/Cu(100)^34^ systems report i* = 1 for *T*_*S*_ = 295 and 213 *K*, respectively, but also emphasize the tendency toward larger i* values for higher deposition temperatures (Ag, Cu) and deposition rates below 2 × 10^−4^ *ML*/*s* (Cu). This tendency is significantly reduced in weakly-interacting film/substrate systems, including Ag and Cu on graphite, for which early STM studies^35–37^ show that dimers are stable at room temperature. This behavior is consistent with the binding energies of ~2 eV for Ag and Cu addimers on graphite, obtained by our DFT calculations (see Table 1; details on the DFT calculation methodology are provided in the “Methods” section) and reported in the literature^38^. Thus, we conclude that the condition i* = 1 is a realistic scenario for the film/substrate systems studied and the growth conditions employed in the present work.

**Table 1:** Values of Ag and Cu adatom adsorption energies *E*_*ads*,*Ag*(*Cu*)_ (atop, bridge, and hollow sites) and Ag_2_ and Cu_2_ addimer binding energies ${E}_{b,A{g}_{2}(C{u}_{2})}$ on the graphene surface calculated via DFT at 0 *K*.

	*E*_*ads*,*Ag*(*Cu*)_ (*eV*)			${{\boldsymbol{E}}}_{{\boldsymbol{b}},{\boldsymbol{A}}{{\boldsymbol{g}}}_{2}{\boldsymbol{(}}{\boldsymbol{C}}{{\boldsymbol{u}}}_{{\bf{2}}}{\boldsymbol{)}}}\,{\boldsymbol{(}}{\boldsymbol{e}}{\boldsymbol{V}}{\boldsymbol{)}}$
	Atop	Bridge	Hollow	
Ag	−0.21	−0.21	−0.20	1.89
Cu	−0.49	−0.49	−0.33	2.21

For a mathematically rigorous calculation of *D*, we take ${{\rm{N}}}_{sat}=\eta {(\frac{F}{D})}^{\frac{2}{7}}$^39^, so that Eq. (2) becomes,

4 $${{\rm{\Theta }}}_{elong}={\eta }^{-\frac{1}{2}}{(\frac{D}{F})}^{\frac{1}{7}}.$$

In Eq. (4), *η* is a proportionality factor that accounts for the dimensionality of the growing islands and i*^39^. *η*(i* = 1) = 0.13, for 3D island growth, and when island saturation density is reached (at Θ ~ 0.4 *ML* according to growth simulation data from ref.^31^). We convert Θ_*cont*_ to Θ_*elong*_ using the ratios *κ* = *Θ*_*perc*_/*Θ*_*cont*_ = *0.5* and 0.3 for Ag and Cu, respectively (see section “Film morphological transitions and evolution”), and the relationship *Θ*_*perc*_/*Θ*_*elong*_ = 1.9, as suggested by Carrey and Maurice for *coalescence-free* growth^23^. By substituting the above ratios to Eq. (4) and solving for *D*, we obtain

5 $$D({T}_{S},F)=F{\eta }^{\frac{7}{2}}{(\frac{\kappa }{1.9}{{\rm{\Theta }}}_{cont})}^{7}.$$

**Figure 4:**
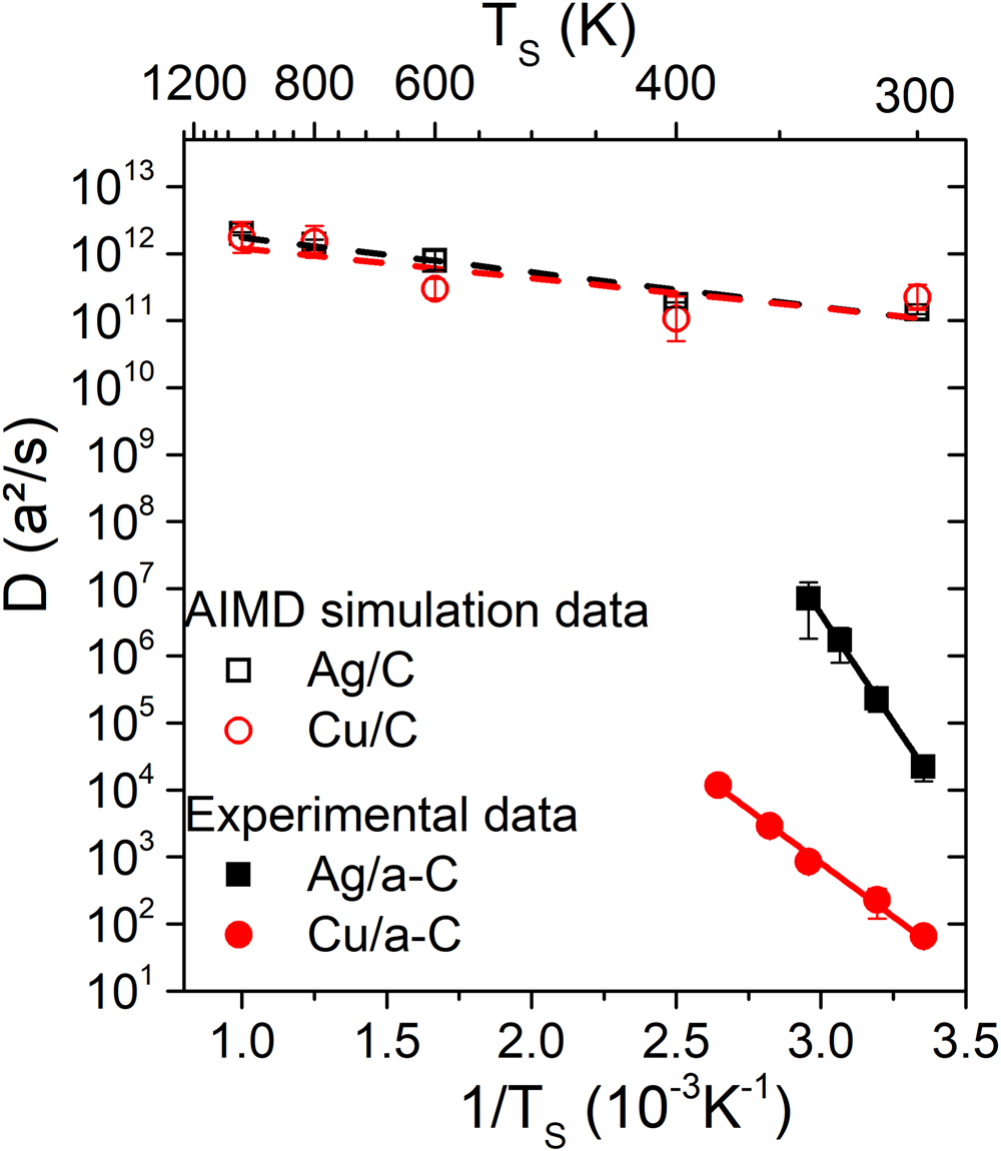
Surface diffusivity *D* as function of temperature *T*_*S*_, as calculated from Eq. (5) from continuous film formation thickness *Θ*_*cont*_ for Ag and Cu diffusion on amorphous carboon (a-C). Hollow symbols correspond to single adatom diffusivity calculated from *ab initio* molecular dynamics (AIMD) simulations (black squares for Ag, red circles for Cu) for diffusion on single-layer graphite. Error bars of the experimental data correspond to the standard deviation after averaging *D*(*F*, *T*_*S*_) over deposition rate *F*, and error bars of AIMD data correspond to the standard deviation of the slopes of adatom mean-square displacements vs. time. Lines represent linear fits of the experimental (full lines) and simulation (dashed lines) data points in the Arrhenius plot.

We then calculate *D*(*T*_*S*_) from Eq. (5), by averaging diffusivities for all *F* values for a given *T*_*S*_.

ln(*D*(*T*_*S*_)) vs. 1/*T*_*S*_ data for Ag (black squares) and Cu (red circles) are plotted in Fig. 4. We find that ${D}_{Ag}^{exp}({T}_{S})$ lies in the range 10^4^ to 10^7^* a*^2^/*s*, with the corresponding values for Cu being between 10^1^ and 10^4^* a*^2^/*s*. In addition, for both Ag and Cu, ln(*D*(*T*_*S*_)) scales linearly with 1/*T*_*S*_, which enables us to calculate the following experimental values for *E*_*D*_ and *ν*_0_: ${E}_{D,Ag}^{exp}=1.28\pm 0.02\,eV$, ${E}_{D,Cu}^{exp}=0.64\pm 0.03\,eV$, ${\nu }_{0,Ag}^{exp}=2.3\,(\,\times \,{2.4}^{\pm 1})\times {10}^{25}\,Hz$, and ${\nu }_{0,Cu}^{exp}=8.2\,(\,\times \,{2.3}^{\pm 1})\times {10}^{11}\,Hz$. Besides the experimental data, Fig. 4 also presents ln(*D*(*T*_*S*_)) vs. 1/*T*_*S*_ values for Ag (hollow black squares) and Cu (hollow red circles) single adatom diffusion on graphite, as determined by AIMD simulations. *D*^*AIMD*^(*T*_*S*_) ranges from 10^11^ to 10^13^*a*^2^/*s* for both Ag and Cu for *T*_*S*_ between 300 and 1000 *K*, which yields $\,{E}_{D,Ag(Cu)}^{AIMD}$ values of 0.10 ± 0.02(0.09 ± 0.04)* eV*, and attempt frequencies ${\nu }_{0,Ag}^{AIMD}$ and ${\nu }_{0,Cu}^{AIMD}$ of 1.4 (×1.5^±1^) × 10^12^ and 8.3 (×2.6^±1^) × 10^11^ *Hz*, respectively. These ${\nu }_{0}^{AIMD}$ values correspond to ${D}_{0,Ag}^{AIMD}$ and ${D}_{0,Cu}^{AIMD}$ of 3.17 (×1.5^±1^) × 10^−3^ and 1.44 (×2.6^±1^) × 10^−3^ *cm*^2^/*s*, respectively.

## Discussion

The up to ten orders of magnitude larger AIMD diffusivities, in comparison to the experimentally-determined values, and the considerably larger ${E}_{D}^{exp}$ vs. $\,{E}_{D}^{AIMD}$ values, indicate that the rate-limiting atomic-scale process that controls early film growth stages and sets Θ_*elong*_ for Ag and Cu on a-C, at the growth conditions employed in the present work, is diffusion of multi-atomic clusters. This is consistent with experimental data showing that Ag clusters are mobile on C-based substrates^40^, even at room temperature It should be noted that in case clusters are mobile, the scaling exponent *y* in the relation ${N}_{sat} \sim {(\frac{F}{D})}^{y}$ is expected to be larger than the value 2/7 for immobile clusters^30^. This would yield a $D \sim {{{\rm{\Theta }}}_{cont}}^{2/y}$ power law with $\frac{2}{y} < 7$ compared to the value $\frac{2}{y}=7$ in Eq. (5). Hence, the diffusivity values plotted in Fig. 4 are to be seen as an upper limit, which means that the conclusions drawn based on the result *D*^*AIMD*^(*T*_*S*_) ≫ *D*^*exp*^(*T*_*S*_) still hold.

Attempt frequencies for metal adatom surface diffusion are typically of the order of 10^12^–10^13^ *Hz*^41^, with our ${\nu }_{0}^{AIMD}$ values being within this range for both Ag and Cu. Compared to single adatoms, clusters may exhibit considerably larger attempt frequencies. For example, Wang *et al*.^42^ have encountered values of ~10^16^ *Hz* for diffusion of *compact* Ir clusters consisting of 19 atoms on Ir(111), while Bardotti *et al*.^43^ found that Au_N_ and Sb_N_ clusters (*N* = 100–1000 atoms) diffuse on graphite surfaces with attempt frequencies of the of the order of ~10^20^ *Hz*. The considerably larger, with respect to adatoms, *ν*_0_ values for clusters reported in refs. ^42,43^. have been interpreted as an effect originating from multiple vibrational degrees of freedom within the cluster, augmented by dynamical mismatch (i.e., the substrate internal vibrations are decoupled from those within the cluster) and weak interactions between the cluster and the substrate^44–46^. Deltour *et al*.^46^ suggested that the propensity of clusters to support internal vibrational states increases with the cluster size. Krylov^45^ used an analytical kinetic model of particle-on-substrate diffusion and showed that surface gliding attempt frequency of an one-dimensional cluster consisting of *N* atoms increases as ~*N*^*α*^, where *α* ≫ 1, while the activation energy increases as ~*N*^*β*^, with *β* < 1. In view of the arguments outlined above, the fact that ${\nu }_{0,Cu}^{exp} < {\nu }_{0,Ag}^{exp}$ may be attributed to differences in cluster size between the two metals, whereby Ag forms larger mobile clusters than Cu. It is also noteworthy that, even though ${E}_{D,Ag}^{exp} > {E}_{D,Cu}^{exp}$, surface diffusivity for the Ag/a-C system is up to three orders of magnitude larger than that for Cu on a-C. This highlights the importance of knowledge of both diffusion barrier and attempt frequency, in order to determine rates of atomic-scale structure-forming processes. This is particularly relevant for film/substrate systems in which adatom diffusion is not the rate limiting step that governs morphological evolution.

## Summary and Outlook

The rates of atomic-scale processes that control the early stages of thin metal film growth on weakly-interacting substrates are not well established in the literature. In the present work, we contributed to the afore-mentioned gap in knowledge by implementing a method suggested recently by Lü *et al*.^14^,—this method utilizes scaling relations of the nominal film thickness Θ at characteristic morphological transitions as a function of deposition temperature *T*_*S*_ and rate *F—*to determine atomic-scale surface diffusion rates during sputter-deposition of Ag and Cu films on a-C substrates.

We determined Θ at percolation (Θ_*perc*_) and continuous film formation (Θ_*cont*_) transition for *T*_*s*_ between 298 and 413 *K*, *F* in the range 0.08 to 5.38 *monolayers*/*s*, from which we estimated the temperature-dependent atomic diffusivity *D*_*Ag*_(*T*_*S*_) and *D*_*Cu*_(*T*_*S*_) on the substrate surface and calculated, experimentally, the following migration energy barriers $$\,{E}_{D}^{exp}$$ and attempt frequencies $${\nu }_{0}^{exp}$$: $${E}_{D,Ag}^{exp}=1.28\pm 0.02\,eV$$, $${E}_{D,Cu}^{exp}=0.64\pm 0.03\,eV$$, $${\nu }_{0,Ag}^{exp}=2.3\,(\,\times \,{2.4}^{\pm 1})$$$$\times {10}^{25}\,Hz$$, and $${\nu }_{0,Cu}^{exp}=8.2(\,\times \,{2.3}^{\pm 1})\times {10}^{11}\,Hz$$. We also performed *ab initio* molecular dynamics (AIMD) simulations, within the framework of density functional theory, and studied diffusion of Ag and Cu adatoms on graphite for *T*_*S*_ = 300–1000 *K*. Analysis of AIMD results yielded adatom migration energy barriers $${E}_{D,Ag(Cu)}^{AIMD}=0.10\pm 0.02\,(0.09\pm 0.04)\,eV$$, and attempt frequencies $${\nu }_{0,Ag}^{AIMD}$$ and $${\nu }_{0,Cu}^{AIMD}$$ of 1.4 (×1.5^±1^) × 10^12^ and 8.3 (×2.6^±1^) × 10^11^ *Hz*, respectively. By critically comparing experiments, simulations and literature data we suggest that: (i) the experimentally-determined diffusivities of Ag and Cu correspond to cluster diffusion, rather than to diffusion of isolated monomers; and (ii) Ag forms larger mobile clusters than Cu on a-C.

The overall results of this work open the way for determining diffusion rates during growth of metals on a wide range of weakly-interacting film/substrate systems. Knowledge of these rates can be used to develop strategies for selectively manipulating atomic processes that drive film morphological evolution, by e.g., use of surfactants^47–49^ or temporally modulated fluxes^9^. Such approaches may, for example, be relevant for directed growth of metals on 2D-material (e.g., graphene and MoS_2_) and oxide (e.g., TiO_2_ and ZnO) substrates, and thereby fabricate high-performance nanoelectronic, catalytic, and optical devices^3,15^.

## Methods

### Film growth

Ag and Cu films were deposited by direct current magnetron sputtering in a high-vacuum chamber (base pressure ~8 × 10^−6^ *Pa*). Ar gas (purity 99.999%) at a pressure of 0.25 *Pa* was used to generate plasma and sputter magnetron sources were equipped with Ag (diameter 7.62 *cm*, purity 99.99%) and Cu (diameter 7.62 *cm*, purity 99.999%) targets. The target-to-substrate distance was 180 *mm*, to minimize radiative heating and energetic bombardment of the film by backscattered Ar atoms, while the angle between substrate and target normal was 25°. Films were grown on Si (100) substrates covered by a 6.5 *nm* thick a-C layer grown *in situ*, prior to Ag and Cu deposition, by sputtering a graphite target (7.62 *cm*, purity 99.995%), with a power of 150 *W*, at an Ar pressure of 0.25 *Pa*. Ag and Cu films were then deposited with growth rates in the respective ranges 0.11 to 5.38 *ML*/*s* and 0.08 to 2.5 *ML*/*s*, set by changing the power applied to the two targets from 5 to 300 *W*. We note that 1 ML corresponds to the distance between (111) crystallographic planes (0.2359 and 0.2089 *nm* for Ag and Cu, respectively). The growth temperature *T*_*S*_ was varied using a resistive heater in the range 298 to 378 *K* for Ag, while the corresponding range for Cu was 298 to 413 *K*. The substrates were heated to *T*_*S*_ and held at this temperature for a period of 1 *h* prior to deposition start. *T*_*S*_ values were confirmed using vacuum-compatible temperature indicators (NiGK Corp.) which change their color irreversibly upon reaching specific temperature (accuracy ±2 *K* below 410 *K* and ±4 *K* above 410 *K*). Deposition rates were determined by *ex situ* x-ray reflectometry (XRR) in an XRD 3000 Seifert diffractometer (line focus Cu source, Ge (220) monochromator selecting K_α1_ Cu radiation). XRR measurements also verified that changing *T*_*S*_ had only minor effects on *F* for a given target power (<4% variation in the *T*_*S*_ range used in the present study).

### Film characterization

**Figure 5:**
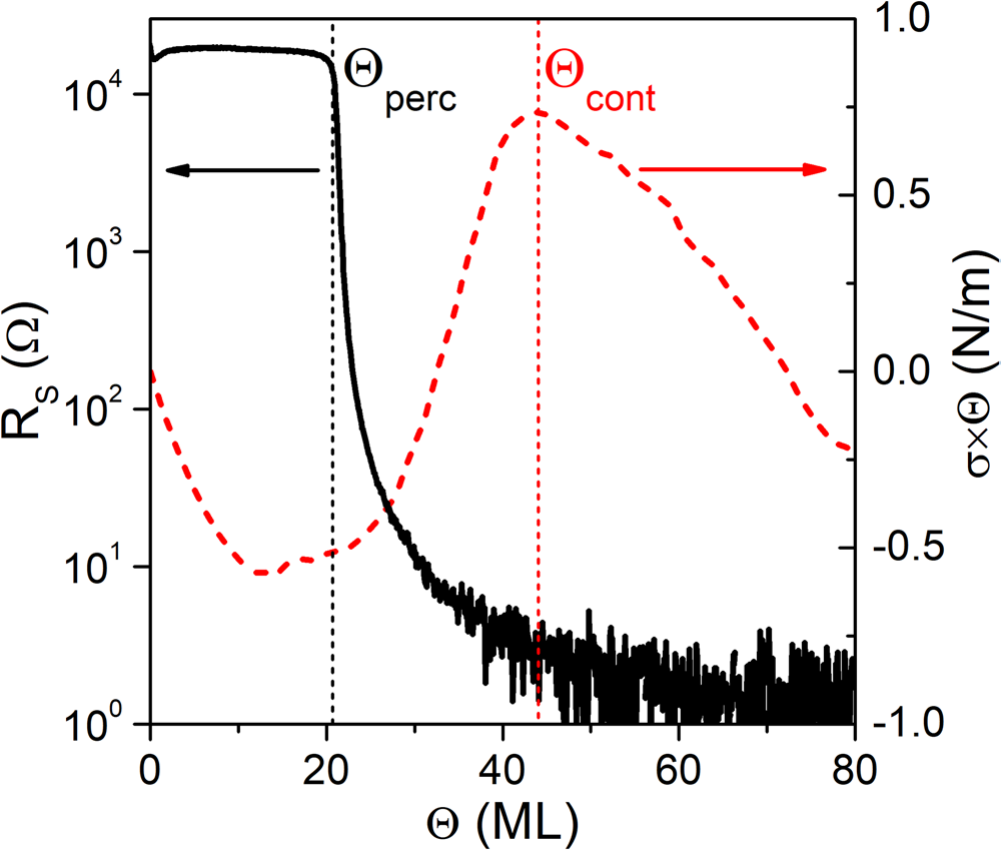
*In situ* and real-time measured evolution of sheet resistance *R*_*S*_ (left axis, black solid line) and stress-thickness product *σ* × *Θ* (right axis, red dashed line) as function of the nominal film thickness *Θ*, for Ag grown on amorphous carbon with deposition rate *F* = 1 *ML*/*s* and at growth temperature *T*_*S*_ = 298 *K*. The abrupt drop in the *R*_*S*_ vs. *Θ* curve at ~20 *ML* marks the percolation transition thickness *Θ*_*perc*_, while the position of the tensile-to-compressive peak in the *σ* × *Θ* vs. *Θ* curve at ~42 *ML* corresponds to the continuous film formation thickness *Θ*_*cont*_.

Θ_*perc*_ was determined, *in situ* and in real-time, by measuring the evolution of the film sheet-resistance *R*_*S*_ vs. Θ with a custom-built four-point probe setup^50,51^ during deposition on a Si (100) substrate (dimension 1 × 1 *cm*^2^, substrate thickness *d*_*s*_ = 350*μm*, resistivity ~20 *k*Ω) covered with a 6.5 *nm* a-C layer. Prior to the film growth, two Au stripes (film nominal thickness Θ = 100 *nm*, width *w* = 2.5 *mm*) were sputter deposited with a Ti adhesion layer (Θ = 40 *nm*,* w* = 2.5 *mm*) onto the a-C layer to ensure a uniform electrical contact. Figure 5 shows a characteristic example of the *R*_*S*_ vs. Θ evolution during Ag deposition on a-C at *F* = 1 *ML*/*s* and *T*_*S*_ = 298 *K* (left axis, black solid line). *R*_*S*_ exhibits a sharp drop at Θ ~ 20 *ML*; this indicates the formation of an electrically conducting film and corresponds to Θ_*perc*_.

Θ_*cont*_ was determined by *in situ* and in real-time measurements of the change of the substrate curvature ${\rm{\Delta }}\kappa$ with a multi-beam optical stress sensor (MOSS, k-Space Associates), described in-detail in refs.^52,53^. Stress measurements were performed for samples grown on Si(100) substrates (*d*_*s*_ = 100 ± 2 *μm*), also covered with a 6.5 *nm* a-C layer. The use of ultra-thin Si(100) wafers allows for a measurement sensitivy of 0.05 *N*/*m*^52^. These measurements enable us to monitor the evolution of the film residual stress *σ*; the film stress-thickness product *σ* × Θ is proportional to ${\rm{\Delta }}\kappa$ via the Stoney equation $\sigma \times {\rm{\Theta }}=\frac{1}{6}{Y}_{s}{d}_{s}^{2}{\rm{\Delta }}\kappa$ (*Y*_*s*_ = 180.5 *GPa* is the Si(100) substrate biaxial modulus)^54^. The slope of the *σ* × Θ vs. Θ curve corresponds to the stress forming in the film, which exhibits a tensile-to-compressive transition (i.e., transition from positive to negative slope) upon reaching Θ_*cont*_ for films with 3D morphological evolution^55–57^. An example for such measurement is given in Fig. 5 (right axis, red dashed line) for Ag deposited on a-C (*F* = 1 *ML*/*s* and *T*_*S*_ = 298 *K*), where the tensile-to-compressive transition is seen at Θ_*cont*_ ~ 42 *ML*. No abrupt curvature change could be detected in the very early stage of metal film growth, confirming the weak interaction between the metal deposit and a-C layer, which is associated with insignificant surface stress evolution. Additional *in situ R*_*S*_ and *σ* × Θ vs. Θ curves are presented in the Supplemental Material^32^.

*In situ* characterization was complemented by *ex situ* imaging of film surfaces using Atomic Force Microscopy (AFM) in tapping mode (Nanoscope III Multimode, Digital Instruments). Surface topography was studied at various film growth stages, and the effect of deposition conditions on film morphology was quantified by calculating the RMS surface roughness. AFM data were processed and analyzed using the WSxM software^58^.

### Density functional theory calculations and molecular dynamics simulations

We used static (i.e., 0 K) DFT to calculate Ag and Cu adatom adsorption energies, as well as Ag_2_ and Cu_2_ addimer binding energies on a-C, i.e., the energy required to dissociate a diatomic (Ag_2_ or Cu_2_) cluster residing on an a-C surface. We approximated the a-C substrate used in the experiments with a 72-atom single-layer graphite (i.e., graphene) sheet. Such choice is motivated by the need of simplifying the computational model and by the fact that short-range order of amorphous carbon surface has been shown to be close to that of graphite, with nearest-neighbor distances of 0.147 and 0.146 *nm* for a-C and graphite, respectively^59^. This length-scale describes the surface that an adatom encounters in its immediate environment. Hence, a similar adatom and addimer adsorption and diffusivity behavior is expected on the two types of surfaces.

DFT calculations were carried out with the VASP code^60^, using the Perdew-Burke-Ernzerhof generalized gradient approximation^61^, and the projector augmented wave method^62^. The approximation proposed by Grimme^63^ was adopted to model the non-locality of electron correlation. 3 × 3 × 1 **k**-point integration of the reciprocal space and 500 eV plane-wave cutoff energies were employed to converge ground-state energies to within an accuracy of 10^−5^ *eV*/*supercell* and minimize forces to less than 0.01 eV/Å. First, we determined the equilibrium lattice parameter and energy *E*_*graph*_ of a 6 × 6 unit cell (72 atoms) graphene sheet. Thus, the adsorption energies *E*_*ads*_ of Ag and Cu adatoms in hollow, atop-C, and bridge (above C–C bond center) positions were evaluated as *E*_*ads*,*Ag*(*Cu*)_ = *E*_*graph*+*Ag*(*Cu*)_ − (*E*_*graph*_ + *E*_*Ag*(*Cu*)_). The energy *E*_*graph*+*Ag*(*Cu*)_ is obtained by adatom vertical relaxation (on the adsorption site) together with full relaxation of C positions. The energies *E*_*Ag*(*Cu*)_ of isolated Ag and Cu atoms were calculated accounting for electronic spin degrees of freedom, using cutoff energies of 870 and 1000 eV, respectively. Ag_2_ and Cu_2_ addimer binding energies *E*_*b*_, determined after full relaxation of two vicinal adatoms in different initial positions on the graphene surface, were calculated as ${E}_{b,A{g}_{2}(C{u}_{2})}={E}_{graph}+2({E}_{ads,Ag(Cu)}\,+\,{E}_{Ag(Cu)})-{E}_{graph+A{g}_{2}(C{u}_{2})}$. DFT results of adsorption and binding energies are summarized in Table 1.

We also used AIMD simulations, within the framework of DFT^64^, to study diffusion of Ag and Cu adatoms. AIMD simulations were based on canonical *NVT* sampling of the phase space, performed by coupling the system with the Nosé-Hoover thermostat, integrating the equations of motion at 1 *fs* timesteps. The dynamics of individual Ag and Cu adatoms on single graphite layers consisting of 72 carbon atoms was modeled at temperatures *T*_*S*_ = 300, 400, 600, 800 and 1000 *K* with total simulation times of ~0.77 *ns* for Ag and ~0.66 *ns* for Cu. Γ-point sampling of the Brillouin-zone and 300 *eV* cutoff energy for the planewave basis set were used for all simulations. Temperature-dependent diffusivities *D*(*T*_*S*_) of Ag and Cu adatoms were computed from the slope of the adatom mean square displacement vs. time according to the methodology suggested by Saxton^65^, from which adatom surface diffusion activation barriers and attempt frequencies were determined via linear regression on *ln*(*D*(*T*_*S*_)) vs. 1/*T*_*S*_ data.

## Supplementary information

Supplemental material
