# Supplementary material for: Atomic-scale diffusion rates during growth of thin metal films on weakly-interacting substrates
Source: Sci Rep. 2019 Apr 29;9:6640. doi: 10.1038/s41598-019-43107-8 (PMC6488595; doi:10.1038/s41598-019-43107-8)
Supplement: Supplementary file 1 — Supplemental material [file 41598_2019_43107_MOESM1_ESM.pdf]

Supplemental material: “Atomic-scale diffusion rates during growth of thin metal films on weakly-interacting substrates”

A. Jamnig[1,2,*], D.G. Sangiovanni[3,4], G. Abadias[1], and K. Sarakinos[2]

*Corresponding author, email: andreas.jamnig@liu.se

[1]*Institut Pprime, Département Physique et Mécanique des Matériaux, UPR 3346 CNRS, Université de Poitiers, SP2MI, 11 Bvd M. et P. Curie, F 86073 Cedex 9, France*

[2]*Nanoscale Engineering Division, Department of Physics, Chemistry, and Biology, Linköping University, SE 581 83, Linköping, Sweden*

[3]*Atomistic Modelling and Simulation, ICAMS, Ruhr-Universität Bochum, D-44801 Bochum, Germany*

[4]*Theoretical Physics Division, Department of Physics, Chemistry, and Biology, Linköping University, SE 581 83, Linköping, Sweden*

We employed *in situ* and real-time sheet-resistance[1] and wafer-curvature measurements[2,3], from which we determined the nominal film thickness Θ at percolation (Θ_{perc}) and continuous film formation (Θ_{cont}) transition, respectively. Θ_{cont} is determined by the nominal thickness at which the slope of the stress-thickness product $\sigma \times \Theta$ undergoes a tensile to compressive transition (i.e., transition from positive to negative slope), while Θ_{perc} corresponds to the thickness at which the film sheet-resistance R_S exhibits a sharp drop indicating the formation of a macroscopically conductive film.

Figure S1 shows representative $\sigma \times \Theta$ vs. Θ curves during growth of Ag on amorphous carbon (a-C) for deposition rates F (a) 0.14 $monolayers/s$ (ML/s), (b) 0.63 ML/s, (c) 5.38 ML/s and temperatures T_S of $298, 313, 354$ and 378 K. We observe that Θ_{cont} increases with increasing T_S, e.g., at $F = 0.14$ ML/s (Fig. S1(a)) Θ_{cont} increases from 50 to 276 ML, when T_S is increased from 298 to 378 K. For constant values of T_S, Θ_{cont} shifts to lower values with increasing F; this effect is more pronounced at the highest temperature $T_S = 378$ K (red dash-dotted line in Fig. S1), where Θ_{cont} decreases from 276 to 115 ML, when increasing F from 0.14 to 5.38 ML/s.

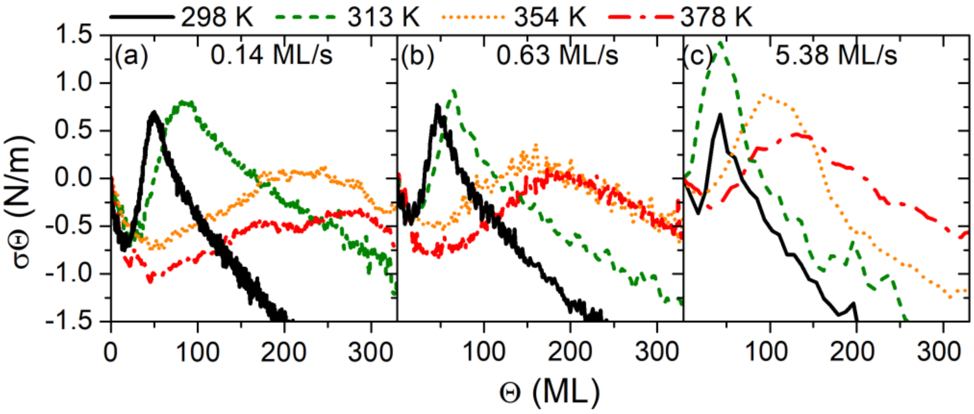

Figure S1. *In situ measured evolution of stress-thickness* $\sigma \times \Theta$ *as function of the nominal film thickness* Θ *for Ag grown on a-C/Si with deposition rate (a)* 0.14 ML/s, *(b)* 0.63 ML/s, *(c)* 5.28 ML/s *and temperatures in the range* 298 *to* 378 K. *The position of the tensile-to-compressive peak in the* $\sigma\Theta$ *vs.* Θ *curves corresponds to the continuous film formation thickness* Θ_{cont}.

Representative R_S vs. Θ curves during Ag growth on a-C are shown in Fig. S2 for $298\,K \leq T_S \leq 378\,K$ and F equal to (a) 0.27 ML/s, (b) 1 ML/s, (c) 5 ML/s. The onset of macroscopic conductivity, i.e., Θ_{perc}, shifts from 26 to 121 ML, when

increasing T_S from 298 to 378 K at F $= 0.27\ ML/s$.(Fig. S2(a)) With increasing values of F, Θ_{perc} shifts to lower thicknesses, e.g., increasing F from 0.27 to 5 ML/s, for $T_S = 378\ K$ (red, short-dotted line in Fig. S2), leads to a decrease of Θ_{perc} from 121 to 77 ML. We note that the R_S values at $\Theta = 0\ ML$ differ among the various measurements. This value primarily depends on the doping of the Si substrate, and for the measurements presented in Fig.S2, different substrate batches were used, which exhibited sheet resistance values spreading over an order of magnitude. These differences, however, do not influence of the quality of the measurements and the resulting Θ_{perc} value.

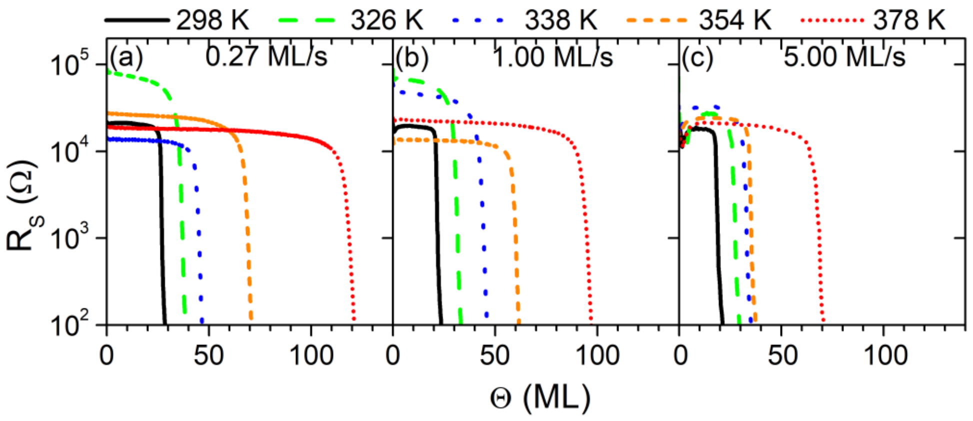

Figure S2. *In situ measured evolution of sheet resistance R_S as function of the nominal film thickness Θ for Ag grown on a-C/Si with deposition rate (a) $0.27\ ML/s$, (b) $1\ ML/s$, (c) $5\ ML/s$ and temperatures in the range 298 to $378\ K$. The position of the sharp drop in the R_S vs. Θ curves corresponds to the percolation transition thickness Θ_{perc}.*

Figure S3 presents $\sigma \times \Theta$ vs. Θ curves during growth of Cu on a-C, for deposition rates F equal to (a) $0.07\ ML/s$, (b) $0.49\ ML/s$, and (c) $2.55\ ML/s$, and T_S values in the range 298 to 413 K. Similar to the results for Ag, we find that Θ_{cont} increases with increasing T_S, e.g., for F $= 0.07\ ML/s$, $\Theta_{cont}(298\ K) = 42\ ML$ and

$\Theta_{cont}(413\,K) = 156\,ML$ (see solid black and dashed-dotted purple lines in Fig. S3(a)). Conversely, Θ_{cont} shifts to lower values for increasing F, e.g., $\Theta_{cont}(0.07\ ML/s) = 156\,ML$ and $\Theta_{cont}(2.55\ ML/s) = 79\,ML$ for $T_S = 413\ \mathrm{K}$ (see dashed-dotted purple line in Figs. S3 (a) and (c)).

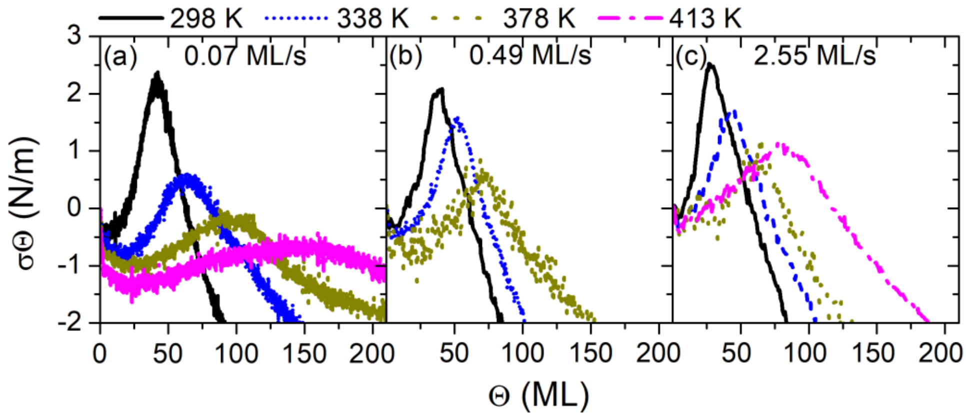

Figure S3. *In situ measured evolution of stress-thickness $\sigma \times \Theta$ as function of the nominal film thickness Θ for Cu grown on a-C with deposition rate (a) $0.07\ ML/s$, (b) $0.49\ ML/s$, (c) $2.55\ ML/s$ and temperatures in the range 298 to $413\ K$. The position of the tensile-to-compressive peak in the $\sigma\Theta$ vs. Θ curves corresponds to the continuous film formation thickness Θ_{cont}.*

Figure S4 presents *ex situ* AFM images for Ag ($T_S = 378\,K,\ F = 5.38\,ML/s$) and Cu ($T_S = 413\,K,\ F = 2.55\,ML/s$) films grown on a-C, at their respective Θ_{cont} values (Figs. 4 (a) and (b)) and for $\Theta\sim 450\ \mathrm{ML}$ (Figs. 4 (c) and (d)). For both thicknesses, Ag surface appears rougher with larger features as compared to Cu. At Θ_{cont}, the root mean-square (RMS) roughness for Ag is $6.55\,nm$, while the value for Cu is $1.66\,nm$. When increasing Θ to $\sim 450\,ML$, the RMS roughness increases to $10.93\,nm$ and $6.22\,nm$ for Ag/a-C and Cu/a-C, respectively, with the value for Cu remaining lower than the respective value for Ag growth. These trends are consistent

with the *in situ* data presented in Figs. S1 and S3, showing that Cu has the tendency to grow flatter and, thus, form continuous layers at lower nominal thicknesses than Ag.

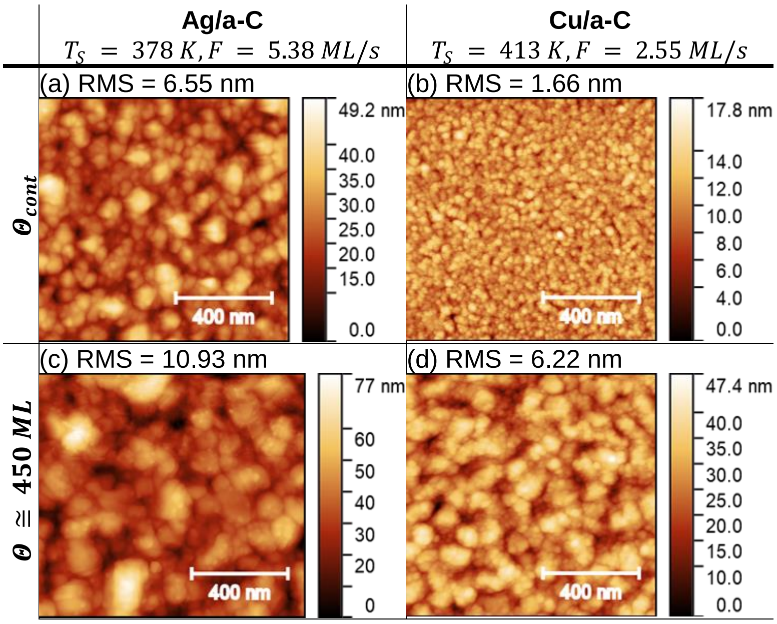

Figure S4. *Atomic force microscopy images of the surface morphology of Ag and Cu grown on amorphous carbon (a-C) with deposition temperature* T_S *and rate* F *indicated. Morphologies are compared for nominal film thickness* Θ *equal to* Θ_{cont} *((a) and (b)) and* $\Theta \cong 450\,ML$ *((c) and (d)). The root mean-square roughness values (RMS) are indicated for the respective films.*

REFERENCES

1. Colin, J. J. *et al.* A load-lock compatible system for in situ electrical resistivity measurements during thin film growth. *Rev. Sci. Instrum.* **87,** 23902 (2016).
2. Chason, E. & Guduru, P. R. Tutorial: Understanding residual stress in polycrystalline thin films through real-time measurements and physical models. *J. Appl. Phys.* **119,** 191101 (2016).
3. Abadias, G. *et al.* Volmer-Weber growth stages of polycrystalline metal films probed by in situ and real-time optical diagnostics. *Appl. Phys. Lett.* **107,** 183105 (2015).
